# Multiple-family group intervention programming to improve mental health of adolescents living with HIV/AIDS in Ghana: An implementation science study protocol

**DOI:** 10.1371/journal.pone.0325854

**Published:** 2025-06-12

**Authors:** Dorothy Serwaa Boakye, Samuel Adjorlolo

**Affiliations:** 1 Department of Health Administration and Education, University of Education, Winneba, Ghana; 2 Department of Mental Health Nursing, School of Nursing and Midwifery, University of Ghana, Accra, Ghana; Public Library of Science, UNITED KINGDOM OF GREAT BRITAIN AND NORTHERN IRELAND

## Abstract

**Background:**

Adolescents living with HIV/AIDS in sub-Saharan Africa face a disproportionate burden of mental health challenges and suboptimal antiretroviral therapy (ART) adherence, which can have significant consequences for their overall health and well-being. While multiple family group therapy (MFGT) has shown promise in improving outcomes for ALHIV in other settings, the intervention is yet to be wholly adapted and evaluated in the Ghanaian context. This study aims to adapt, pilot, and evaluate a multiple family group therapy (MFGT) intervention to improve mental health and ART adherence among adolescents living with HIV/AIDS in Ghana.

**Methods:**

A total of 80 ALHIV (aged 10–19 years at enrollment) will be recruited from two HIV clinics and each clinic will be randomized to one of two study conditions (MFGT group and control group). The study will be conducted in three phases: 1) Adaptation Phase – Qualitative data collection through focus group discussions and in-depth interviews with key stakeholders (adolescents, caregivers, community leaders and healthcare providers) to inform the adaptation of the MFGT intervention for the Ghanaian context; 2) Pilot Implementation Phase – Quantitative assessments (PHQ-9, GAD-7, Wilson’s 3-item adherence scale, FAD, Berger HIV Stigma Scale) and qualitative data collection (focus groups, interviews) to evaluate the feasibility, acceptability, and preliminary effectiveness of the adapted MFGT intervention; and 3) Evaluation Phase – Continuation of the quantitative and qualitative data collection, as well as a cost-effectiveness analysis, to assess the long-term impact, sustainability, and scalability of the MFGT intervention.

**Expected Outcomes:**

The study will provide valuable insights into the feasibility, acceptability, and effectiveness of the adapted MFGT intervention in improving mental health and ART adherence among adolescents living with HIV/AIDS in Ghana. The findings will inform the development of culturally relevant and sustainable strategies to support this vulnerable population, with potential implications for similar interventions in other sub-Saharan African contexts.

**Ethics and Dissemination:**

The study has obtained necessary ethical approvals from the Ghana Health Service Ethics Review Committee and relevant health authorities, and will follow international research guidelines. Findings will be disseminated through peer-reviewed publications, conference presentations, and clinical trial registry, with an emphasis on open-access formats to maximize accessibility.

**Protocol Registration in Clinical Trials.gov:**

(NCT06701942).

**Date of trial registration:**

24 November, 2024.

## 1. Introduction

Globally, 38.4 million people are living with HIV, of which 1.7 million are children aged 0–14 years [1]. Sub-Saharan Africa contributes substantially to the HIV burden, reflected in the high HIV prevalence, mortality and morbidity [2]. Relating to Ghana, about 1.7% of population is living with HIV, with adolescents aged 15–19 years contributing to 0.7% of the HIV burden [3]. The diagnosis of HIV potentiates a cascade of mental health problems that impact all aspect of HIV prevention, treatment/management. This vulnerability is significantly elevated in adolescents who have heightened risk for mental health problems owing to their developmental stage [4]. In a recent global meta-analytic study, the prevalence of depression among ALHIV was 26.07%, with females (32.15%) and older adolescents aged 15–19 years (37.09%) recording the highest rate than males (25.07%) and younger adolescents aged 10–14 years (29.82%), respectively [5].

Focusing on SSA, a recent systematic review found that 25% of ALHIV screened positive for any psychiatric disorder, whereas 30–50% presented with emotional, behavioral difficulties or significant psychological distress [6]. Despite the paucity of epidemiological studies in Ghana, existing evidence suggests that ALHIV suffer from discrimination and rejection, ushering them into profound psychological distress such as suicidal thoughts and hopelessness [7] that often culminate into poorer psychosocial adjustment [8]. Unaddressed mental health problems in ALHIV contribute to not only mental health burden but also negatively impact on treatment adherence, prevention of HIV through the adoption of safe sexual practices and behaviors, school attendance and academic achievement [9].

In view of the above, analysts have called for a proactive response to the mental health issues in ALHIV [10]. In particular, the healthcare systems has been admonished to provide integrated healthcare to ALHIV that includes mental health [6,11]. Doing so would not only strengthen HIV prevention and care outcomes, but it would additionally improve global access to mental healthcare [9]. Indeed, living with HIV as an adolescent raises the exigency for effective support and guidance, including addressing mental health issues, to ensure they traverse through this developmental stage [12]. However, the literature is replete with the general neglect of mental health problems in ALHIV, particularly in low-resource settings where investment in and delivery of mental health are notably poor and disorganized [13]. Significant advances have been made in the prevention and treatment of HIV. For example, adolescents diagnosed with HIV can expect a near normal life span given access and adherence to combination antiretroviral therapy (cART) [14,15]. Ghana, like other countries, has subscribed to the Joint United Nations Programme on HIV/AIDs goal of 95-95-95 call for 95% of people living with HIV (PLWH) to be diagnosed, with 95% of them initiating cART, and 95% of those on cART to achieve and sustain viral suppression through adherence to the treatment. However, these gains will not be achieved without addressing the significant mental health and substance use problems among ALHIV [9]. The lack of research and support for mental health needs in Ghana and other resource-limited settings presents an enormous burden for which cost-effective solutions are urgently needed [11].

Owing to the limited human resources, finance and logistics, the healthcare system in Ghana, just like other countries in SSA, is fragmented mostly at the primary level. Mental health services are barely integrated into the primary healthcare for ALHIV. Most of the psychiatric units of the Ghanaian hospitals are poorly resourced, making referrals of ALHIV in need of mental support to psychiatric hospitals the most viable option. However, financial problems, geographic barriers and others limit the utilization of the referral system, leaving the mental health of ALHIV unaddressed. This demands the integration of the mental health support system into the primary care for ALHIV; the multiple family group therapy (MFGT) holds significant prospects in this regard.

This study proposes Multiple Family Group Therapy (MFGT) as a potential solution to address these challenges. By bringing together multiple families affected by HIV, MFGT provides a platform for shared learning, support, and skill development that aligns with both mental health and HIV treatment goals. The present study aims to adapt, implement, and evaluate an MFGT intervention for ALHIV and their families in Lower Manya Krobo, Ghana. This research addresses critical gaps in the literature regarding culturally adapted mental health interventions for ALHIV in resource-limited settings. It provides evidence for integrating mental health support into primary HIV care. The findings will contribute to developing sustainable, scalable solutions for improving both mental health and HIV outcomes among adolescents in Ghana and similar settings.

## 2. The multiple family group therapy

Multiple Family Group Therapy (MFGT) is a cost-effective intervention that holistically addresses psychosocial challenges among adolescents living with HIV (ALHIV) and their families. Listed on the U.S. National Registry of Evidence-based Programs, MFGT uses minimal resources to achieve positive mental health outcomes [16]. The program focuses on the “4Rs” (Rules, Responsibility, Relationship, and Respectful communication) and “2Ss” (Stress and Social support), aiming to foster mutual support and learning among families facing similar challenges [17].

Originally developed for children with behavioral disorders, MFGT has been successfully adapted to address various mental health issues, including depression and anxiety in HIV-affected populations. The intervention combines group and family therapy approaches, supporting healthcare engagement, treatment adherence, symptom reduction, and improved quality of life. While based on Western therapeutic principles, MFGT has shown effectiveness when facilitated by lay counselors and community health workers [18,19], making it suitable for low-resource settings like Ghana.

However, implementing MFGT in Ghana presents two main challenges. First, the program’s Western, individualistic approach contrasts with Ghana’s collectivist family systems, necessitating cultural adaptation [16,20]. Second, while versions of MFGT have been used in family-based HIV prevention and economic strengthening programs in other African countries [18,19,21], this represents the first attempt to fully adapt MFGT for addressing mental health issues among ALHIV in sub-Saharan Africa, specifically in Ghana. This adaptation aims to strengthen the healthcare system’s capacity to provide integrated mental health support while respecting local family structures and cultural practices.

## 3. Theoretical frameworks guiding the adaptation, implementation and evaluation phases

*The Theory of Triadic Influence* (TTI) [22] will guide the MFG intervention’s cultural and contextual adaptation process for the Ghanaian context. The TTI is a comprehensive framework that integrates multiple factors influencing health-related behaviors. It organizes these factors into three interacting streams of influence, each operating across varying levels of control (proximal, distal, and ultimate) to explain how health behaviors are shaped and changed. The TTI recognizes that childhood behavioral problems arise from several influencing streams: an intrapersonal stream connected to the child’s inherent traits, a social normative stream shaped by the family environment and the child, and a cultural attitudinal stream influenced by the broader sociocultural context [23]. A review by Flay, Petraitis and Hu [24] highlights that TTI is well-suited for understanding and addressing health behaviors influenced by multiple interacting factors, including individual attitudes, family dynamics, and broader cultural contexts. In a study on the cultural adaptation of a family-based intervention for Latino adolescents, Pantin, Prado [25] used TTI to adjust the content of the intervention by targeting the multiple levels of influence, ensuring that personal and family-level factors were equally addressed. A study by DiClemente, Murray [26] demonstrated that culturally tailored interventions based on the TTI were effective in addressing HIV prevention among African American adolescents. Similarly, adapting MFGT for adolescents in Ghana can benefit from TTI’s structure, which allows for the incorporation of local cultural norms and family structures. A study by Doku, Dotse and Mensah [27] in Ghana showed that family support plays a crucial role in medication adherence among adolescents living with HIV, further supporting the need for a multilevel approach like TTI that addresses both personal and familial influences.

We will complement the TTI with models from *Social Action Theory* [28], a framework for understanding behavior change. Social Action Theory posits that behavior is influenced by the individual’s self-regulatory capabilities, the social environmental context, and the interactions between the individual and the environment. *The adaptation coding framework* developed by Stirman, Miller [29] will guide the documentation of the modifications made to the 4Rs and 2Ss components of the MFG intervention to ensure cultural and contextual relevance for the Ghanaian setting. The framework specifies who is responsible for making the modifications, what aspects are modified, the delivery level, the types of contextual adjustments, and the nature of the content modifications.

*The Family Systems Theory* will guide the pilot implementation phase of the study. Family Systems Theory emphasizes that individuals are best understood within the context of their family unit, making it highly relevant for adolescents living with HIV in Ghana. Family support plays a critical role in managing the illness, and by applying this theory, Multiple Family Group Therapy (MFGT) can address how family beliefs, behaviors, and communication patterns impact the adolescent’s mental well-being and treatment adherence [30], 2012). Unresolved conflicts or poor communication within the family can hinder the adolescent’s mental health and ability to manage their condition. The MFGT delivery, guided by this theory, promotes healthier family interactions, encouraging all members—parents, siblings, and extended family—to provide emotional and practical support [31]. This approach is particularly effective in Ghana’s context, where extended family structures and collective responsibility are valued [27]. The theory also fosters family resilience by encouraging adaptable roles and problem-solving strategies, helping families cope with the challenges of HIV and ensuring long-term success in the adolescent’s health outcomes [32]. The MFGT’s applicability to diverse cultural contexts, such as in Ghana, makes it an ideal theoretical foundation for MFGT’s implementation.

*The RE-AIM framework*, a comprehensive model used to evaluate public health interventions, will guide the evaluation phase. It provides a structured approach to assess the overall impact of an intervention across five key dimensions: Reach, Effectiveness, Adoption, Implementation and Maintenance [33]. The RE-AIM framework has been widely adopted for planning and evaluating public health interventions, particularly in high-income countries [34]. It has shown utility in assessing community-based physical activity programs [35] and evidence-based initiatives for older adults [36]. A systematic review of multi-level obesity-related interventions found that those evaluated using RE-AIM demonstrated potential for broad reach, effectiveness, adoption, and maintenance [37]. However, inconsistent data availability and reporting can hinder comprehensive assessment across all RE-AIM dimensions [34,35]. Despite these challenges, RE-AIM remains a valuable tool for evaluating the public health impact of interventions, particularly when applied using mixed-method approaches [35,37].

## 4. Methods

**Fig 1:**
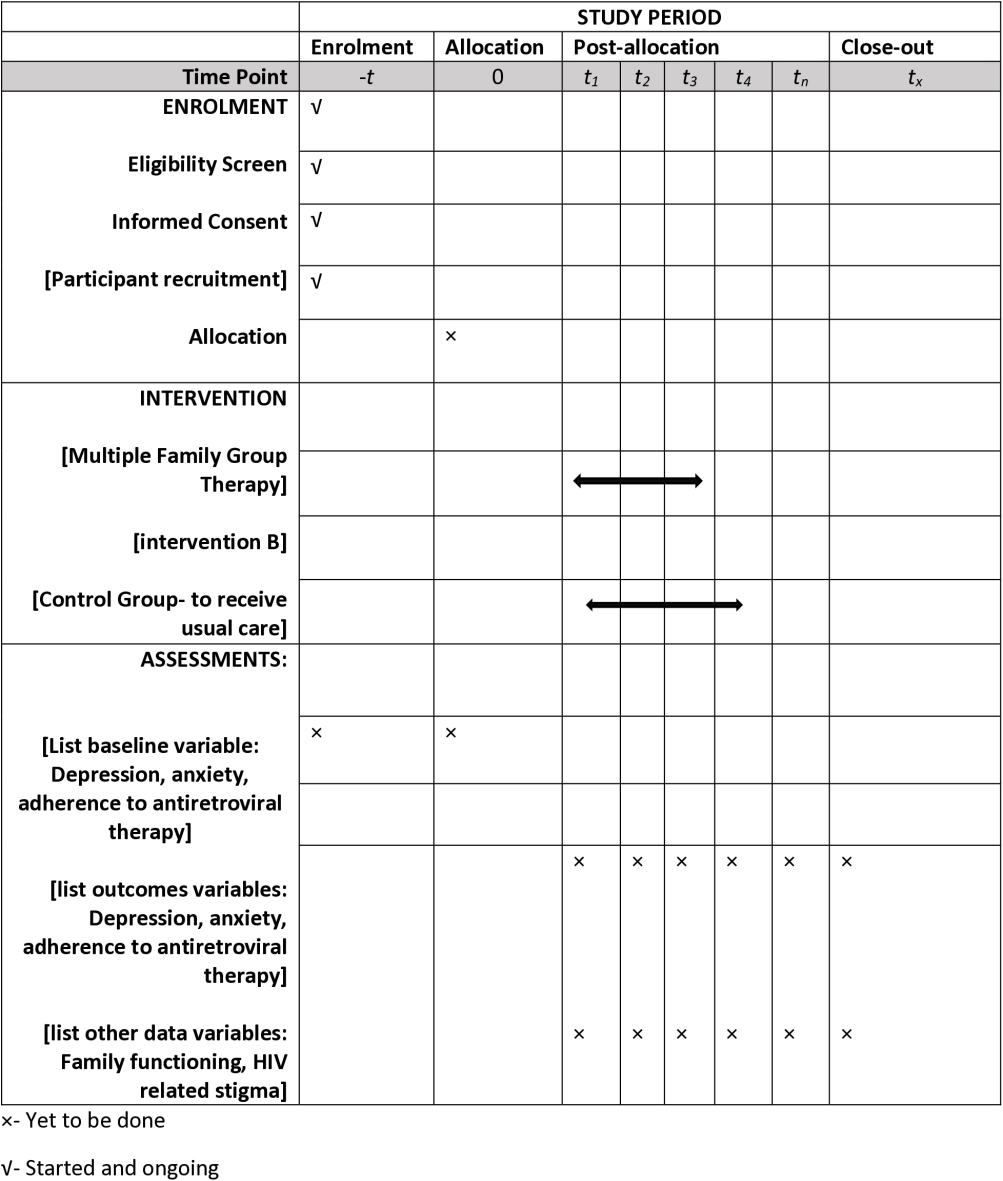
Schedule of study implementation.

This study protocol follows the Standardized Protocol Items Recommendations for Interventional Trials (SPIRIT) guidelines to ensure comprehensive and structured reporting. Complete SPIRIT checklist and WHO Trial Registration Data Set are provided in the supporting materials S1 and S2 Files. Details of study timelines are also provided in the SPIRIT template (Fig 1).

### 4.1. Study setting

The study would be carried out in the Atua and Asesewa Government health facilities within the Lower Manya-Krobo district. The area has a youthful population structure, with a significant proportion under 20 years old [38]. Lower Manya Krobo has historically had one of the highest HIV prevalence rates (5.6%) in Ghana, significantly above the regional (2.8%) and national average (1.6%) [39,40]. The area has strong traditional cultural practices, including the dipo rites of passage for young women, which can influence health behaviors and attitudes towards HIV/AIDS [41]. This setting provides a crucial context for studying HIV/AIDS interventions due to its high prevalence rates and the complex interplay of cultural, economic, and social factors that influence health outcomes for adolescents living with HIV/AIDS and the presence of established health facilities.

### 4.2. Study design and Philosophical underpinning

The study will adopt a cluster randomized controlled trial design, where participating clinics will be randomized to either the intervention arm (MFGT plus usual care) or control arm (usual care only). This design, combined with a mixed-method participatory approach, aligns with the study’s pragmatic philosophical underpinnings. This comprehensive methodology allows researchers to leverage multiple sources of evidence to address the research questions while generating robust insights to inform the design and implementation of the MFGT intervention. The mixed-methods design particularly strengthens the understanding of both intervention effectiveness and implementation processes within the real-world clinical setting.

### 4.3. Study population

Adolescents living with HIV and their primary caregivers/families (at least two generations).

#### 4.3.1. Inclusion criteria.

Adolescents aged 10–19 years (as per WHO definition of adolescence) with confirmed HIV-positive status, aware of their HIV status and currently receiving antiretroviral therapy (ART). Primary caregiver of an eligible adolescent participant aged 18 years or older and aware of the adolescent’s HIV status. Additional criteria include adolescents and their caregivers residing in Lower Manya Krobo district, able to communicate in either English or the local language and willing to participate in group sessions.

#### 4.3.2. Exclusion criteria.

Adolescents and caregivers/families with severe cognitive impairment that would prevent participation in group activities and/ or acute psychiatric conditions requiring immediate intensive treatment, Adolescents and caregivers planning to relocate outside of Lower Manya Krobo district within the next 12 months, unable to commit to attending the majority of planned group sessions, adolescents participating in another mental health intervention study and unable to provide assent or obtain caregiver consent. Caregivers unable to provide informed consent will also be excluded.

## 5. Study phases

### 5.1. Adaptation phase

The study will begin with an adaptation phase focused on culturally adapting the existing MFG (Multi-Family Group) intervention to the local context of Lower Manya Krobo district. This phase is critical to ensure the intervention’s relevance, acceptability, and alignment with the cultural norms and practices of the target population. The adaptation process will involve a four-phase stakeholder engagement and collaborative meetings, guided by previous adaption efforts [16,20,42] and theories of adaption [22,28,29]. Phase 1 is mapping and recruitment of at least 30 stakeholders and initial meetings with stakeholders to create awareness about the project, solicit their views on the scope and expectations of the project and empower them as agents of change to facilitate sustainable collaborations and scale-up of the intervention. Therefore, the powers and influence of each stakeholder is critical to the project. The stakeholders will comprise parents, older family members (e.g., Nephew, Cousin), children/adolescents, family heads, local chiefs and queen mothers, health professionals, including community health officers. The MFGT, focusing on 4Rs and 2Ss, would be presented to the stakeholders to discuss their relevance and application in Ghanaian families. Phase 2 involves a review of the MFGT curriculum/manual. Prior to this, the PI and the research team, with the support of supervisors and other local experts will review the MFGT manual to understand the relevance of its concepts and content for the Ghanaian context. The team will also brainstorm about context-specific activities and examples to be adapted or added. During the review, the stakeholders will be encouraged to note the aspect of the MFGT they have issues with and how the issues can be addressed. These will be discussed with all stakeholders present to arrive at a solution. The stakeholders will address questions such as the number of families for the MFGT, family composition, the timing of intervention delivery, duration and number of sessions, and their opinions about who should lead intervention delivery (i.e., health professionals versus parent peers), location of intervention delivery, the target behaviors for intervention and expected outcome and assessment of outcome. Phase 3 is revision of the MFGT curriculum content based on the inputs, suggestions, and feedback from the stakeholders in phase 2. This is the responsibility of the PI and research team, with the support of project supervisors. Phase 4 is finalization and approval of adapted curriculum/manual. The stakeholders would be invited to review the revised curriculum, ensuring that the content captures all discussions and issues raised in phase 2. The request for new revisions would be tolerated if the majority of the stakeholders endorse the revision. This iterative process would be followed until the stakeholders accept the adapted curriculum as reflective of and sensitive to Ghanaian culture and family value systems.

### 5.2. Pilot implementation phase

The pilot implementation phase involves implementing the culturally-adapted MFGT intervention in selected healthcare facilities in Lower Manya Krobo district. This phase includes training healthcare providers and parent peers, recruiting adolescents with HIV/AIDS and their families, conducting structured group sessions, and monitoring the implementation process. Key session topics cover family roles, communication, problem-solving, and HIV management. This pilot phase aims to assess the feasibility, acceptability, and initial effectiveness of the adapted intervention in the local context.

#### 5.2.1. Facilitator training and qualifications.

The MFGT intervention will be facilitated by a combination of parent peers and community health workers, bringing together lived experience and professional expertise [43,44]. Parent peers, who have personal experience with HIV/AIDS care in the local community, offer unique insights and deeper participant connections [45]. Research demonstrates that such peer interventions effectively improve health outcomes [46]. Community health workers must have a minimum diploma or bachelor’s degree in relevant fields, two years of experience with HIV-affected families, and fluency in English and Dangbe. All facilitators will complete a 5-day intensive training workshop covering intervention content, facilitation skills, cultural competence, and ethical considerations [44,47] specific to the Krobo context. The training will be followed by ongoing supervision, including weekly group meetings and individual support as needed, to ensure quality implementation and fidelity to the adapted intervention while maintaining cultural sensitivity. This comprehensive approach to facilitator selection, training, and supervision aims to create a strong foundation for effective program delivery that balances professional expertise with cultural understanding and peer support [43,44].

#### 5.2.2. Participant recruitment.

The recruitment process will involve collaboration between the research team and local healthcare facilities (Atua and Asesewa). Healthcare providers at HIV/AIDS clinics will be trained to identify eligible adolescents and families during routine visits, while community health workers and HIV support groups will help raise program awareness. Initial contact will be made during clinic visits, where staff will provide a program overview to interested families. The research team will then conduct follow-up meetings for consent and enrollment, alongside group information sessions at clinic sites. All information will be provided in both English and local language (Dangbe) to ensure accessibility. This multi-faceted recruitment approach aims to leverage existing healthcare infrastructure while ensuring comprehensive program information reaches potential participants through various channels.

#### 5.2.3. Screening and final enrollment into the study.

Interested participants will undergo screening based on established inclusion/exclusion criteria and mental health assessments. The screening process helps identify those needing immediate mental health referrals and confirms program suitability. Eligible families who wish to participate will complete the informed consent process, with written consent obtained from caregivers and written assent from adolescents, ensuring full understanding of the program and voluntary participation.

#### 5.2.4. Randomization and allocation.

**Fig 2:**
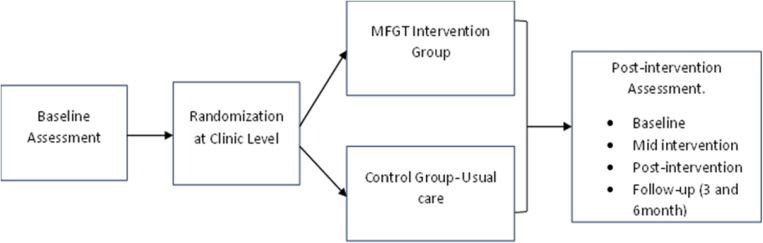
Randomization process and group allocation.

The study will employ a cluster-randomized controlled trial design, where participating clinics, rather than individual participants, will be randomized to intervention or control groups. This approach minimizes contamination between groups and strengthens study validity [48]. Clinic allocation will be determined using a computer-generated randomization sequence to ensure unbiased assignment and concealment [49] (see Fig 2).

#### 5.2.5. Sample size calculation.

The sample size is determined based on the practical difference in expected mean (pre- post-intervention outcome) in the intervention arm. Using the G*power2 computer program [50], a significance level (α) of 0.05, power of 80% (β = 0.2), and an allocation ratio of 1:1 for two independent arms of the study, a total of 72 ALHIV is needed to detect an effect of 0.6 (approximately 36 per study arm). We anticipate an attrition/non-response rate of 10% for each arm of the study (i.e., 10% of 36 = approximately 4 additional participants for each study arm). Therefore, a minimum of 80 ALHIV and their families would be recruited for the study, with each study arm requiring a minimum of 40 participants. Previous studies that adopted versions of the MFGT in South Africa included 12–20 families [19]. Therefore, barring no change in the number of families for an MFGT in the Ghanaian context, a minimum of four MFGTs and a maximum of two MFGTs for each study arm would be needed to achieve a sample size of 80.

#### 5.2.6. Intervention group and description of the intervention conditions.

The clinics allocated to the intervention group will receive the Multiple-Family Group Therapy (MFGT) intervention in addition to the usual care. All eligible and consenting adolescents living with HIV/AIDS and their families who are associated with this clinic will be assigned to the MFGT intervention condition. The MFGT intervention will involve a series of structured group sessions facilitated by parent peers and community health workers trained by the research team. ALHIV and their families will be invited to participate in 16 program sessions, each lasting 90 minutes, and will be held on clinic days over 14 weeks. The MFGT will combine group discussions and activities fostering support, learning and interaction both within the family and among families.

Sessions on the MFGT will begin with introducing participants to one another, the facilitators, and the program curriculum. This initial session is designed to help participants start building connections and becoming familiar with each other. It will also establish the foundation for future sessions by setting group ground rules and determining the meeting location and schedule. Subsequent sessions will concentrate on areas such as respectful communication, support and resilience building, problem-solving, conflict resolution, stress management, and enhancing family relationships and practices. Additionally, to tackle stigma stemming from societal myths about HIV/AIDS, a session may be included to provide adolescents and their families with behavioral health knowledge, fostering discussions about stereotypes, stigma, and their effects on mental health and adherence to ART.

#### 5.2.7. Control group and description of the control conditions.

The clinic allocated to the control group will continue to provide standard HIV care and treatment services. All eligible and consenting adolescents living with HIV/AIDS and their families who are associated with the control clinic will be assigned to the “treatment as usual” condition which includes adherence support from community adolescent treatment supporters, adherence counselling from health workers, Nutritional counselling, health education, treatment of opportunistic infections, medical care and mobile reminders of schedule hospital appointment and treatment adherence. Other services will include defaulter tracing and loss to follow-up services. This control group will allow the research team to compare the outcomes of the adolescents and families receiving the MFGT intervention to those who continue to receive the standard HIV care, thereby evaluating the effectiveness of the MFGT program.

#### 5.2.8. Criteria for discontinuing or modifying allocated interventions.

Participants in the intervention group (MFGT plus usual care) may discontinue the program for several reasons, including voluntary withdrawal at any time without penalty, medical reasons if participation causes significant distress or interferes with treatment, safety concerns if participation poses risks to the participant or others, or extended absence such as missing three consecutive sessions without communication. The intervention may be modified through alternative scheduling or individual make-up sessions for those unable to attend regular meetings, additional support or modified activities for participants experiencing specific challenges, or adjustments to group dynamics and composition while maintaining core intervention components. All discontinuations and modifications will be documented and reported in the study findings, and participants who discontinue MFGT will continue to receive usual care through their clinic.

### 5.3. Evaluation phase

The final phase of the study is the evaluation phase, which focuses on assessing the effectiveness of the culturally-adapted MFGT intervention. The evaluation phase of the study will be iterative and formative in nature [51]. Attention will be paid to both the effectiveness of the MFG intervention and the participants’ experiences throughout the program [52]. The evaluation will be achieved in three steps:

#### 5.3.1. Step 1: Process evaluation.

The process evaluation aims to assess the MFGT intervention’s implementation and identify potential improvements [53,54]. This evaluation will be conducted through session observations, facilitator and participant interviews, and review of attendance records, with findings used to optimize the program for the Ghanaian context.

The evaluation will focus on four key aspects: reach (examining enrollment, attendance, and retention rates), quality (assessing program materials and delivery components), implementation fidelity (monitoring adherence to planned activities), and participant satisfaction (gathering feedback through surveys, interviews, and focus groups). This comprehensive evaluation approach ensures that all aspects of program delivery are examined while identifying areas for refinement and improvement.

#### 5.3.2. Step 2: Impact evaluation.

The impact evaluation will assess the MFGT intervention’s immediate effectiveness through the use of standardized questionnaires. This phase will assess the immediate changes in the targeted outcomes (depression, anxiety, and ART adherence). This comprehensive evaluation approach will help determine whether the intervention has achieved its intended objectives and produced meaningful changes in participants’ mental health and treatment outcomes.

#### 5.3.3. Step 3: Outcome evaluation.

The final evaluation phase will assess the MFGT intervention’s long-term effects. Long-term effects will be measured at 3 and 6 months post-intervention, focusing on sustained improvements in mental health outcomes and ART adherence. This longitudinal assessment will help determine the program’s lasting impact on participants’ health status and quality of life [54].

#### 5.3.4. Study hypothesis.

We hypothesize that;

1. ALHIV in the intervention arm (MFGT group) would have a significant reduction in depression and anxiety and improved adherence to ART, compared to ALHIV in the control condition.
2. Two variables- family functioning and HIV-related stigma will mediate the effects of the MFGT intervention on the primary outcomes (Depression and Anxiety) and the secondary outcome (Antiretroviral therapy (ART) adherence).

#### 5.3.5. Primary outcome measures.

The study’s primary outcome is the improvement in mental health and overall well-being of adolescents living with HIV/AIDS, specifically focusing on changes in depression and anxiety symptoms. Depression will be measured using the Patient Health Questionnaire (PHQ-9), a validated 9-item self-report tool that evaluates symptom presence and severity [55]. The PHQ-9, validated for use with Ghanaian adolescents [56], uses a 4-point scale (0–3) with total scores ranging from 0–27, categorizing depression as moderately severe (10–14), severe (15–19), or very severe (20–27).

Anxiety will be assessed using the Generalized Anxiety Disorder (GAD-7) scale, a 7-item self-report measure also validated for Ghanaian adolescents. The GAD-7 uses a similar 4-point scale with total scores ranging from 0–21, categorizing anxiety as moderate (10–14) or severe (15–21). A cutoff score of ≥10 indicates moderate to severe anxiety symptoms, providing a clear metric for evaluating the MFGT intervention’s impact on participants’ mental health [57,58].

#### 5.3.6. Secondary outcome measure.

Adherence to antiretroviral therapy will be measured as a secondary outcome using the validated Three-Item Self-Report Measure for Medication Adherence [59]. This tool assesses medication adherence through three questions: the percentage of HIV medication taken in the last 4 weeks (0–100% scale), the number of days missed taking medication in the last 4 weeks, and a self-evaluation of adherence quality (5-point Likert scale from “very poor” to “excellent”). These combined measures provide a comprehensive assessment of ART adherence, helping establish the relationship between mental health outcomes and HIV treatment compliance, thus supporting the rationale for implementing the MFGT intervention.

#### 5.3.7. Mediators.

In addition to the primary and secondary outcome variables, the study will also examine potential mediating factors (Family functioning, HIV-related stigma) that may influence the relationship between the MFG intervention and the desired outcomes.

Family functioning will be measured using the McMaster Family Assessment Device (FAD), a 60-item self-report instrument evaluating six domains: problem-solving, communication, roles, affective responsiveness, affective involvement, and behavior control [60]. The FAD has been validated across multiple African contexts, including South Africa, Ethiopia, and Uganda, making it suitable for assessing family dynamics before, during, and after the MFGT intervention [61–63].

HIV-related stigma will be assessed using the Berger HIV Stigma Scale, which measures enacted, internalized, anticipated, and vicarious stigma [64]. The Berger HIV Stigma Scale (HSS) is a widely used measure of HIV-related stigma, demonstrating good reliability and validity across diverse populations [65] and the African context [66]. The scale has been recently validated in Ghana, resulting in a 34-item version with high reliability (α = 0.808) and maintaining the original scale’s four-factor structure [67]. This validated tool will provide a culturally appropriate measure of HIV-related stigma among adolescent participants in Ghana.

#### 5.3.8. Data collection.

The data collection process for this study will be executed in three distinct phases.

##### 5.3.8.1. *Adaptation phase.*

The adaptation phase will utilize focus group discussions (FGDs) and in-depth interviews to gather qualitative data essential for adapting the MFGT intervention to the Ghanaian context. FGDs will be conducted with groups of 6–8 participants, including ALHIV, their caregivers, and healthcare providers. Additionally, in-depth interviews will be held with 10 key stakeholders, including policymakers, community leaders, and experts in HIV/AIDS and adolescent mental health, to understand broader contextual factors and potential implementation challenges.

##### 5.3.8.2. *Pilot implementation phase.*

We will gather qualitative data through focus group discussions and in-depth interviews to gain deeper insights into feasibility, acceptability, and perceived effectiveness of the adapted MFGT program. The FGDs will explore the participants’ experiences, perceptions, and feedback on the adapted MFG intervention.

##### 5.3.8.3. Evaluation phase.

The evaluation will include quantitative assessments of primary outcomes (using PHQ-9 and GAD-7 scales), secondary outcomes (ART adherence using Wilson’s 3-item scale), and mediating factors (McMaster Family Assessment Device and Berger HIV stigma scale) at multiple time points: baseline, post-intervention, 3-month follow-up, and 6-month follow-up. Qualitative data will be gathered through focus group discussions with adolescents, caregivers, and program facilitators to understand the intervention’s long-term impact and sustainability.

#### 5.3.9. Data management and analysis plans for quantitative and qualitative data.

The data entry will be conducted by an independent staff member not involved in the research team, who will input data into separate datasheets. This ensures that researchers can conduct analyses while remaining blinded to group allocation. The quantitative analysis will involve descriptive statistics at all time points, with repeated-measures ANOVA examining changes in primary and secondary outcomes across baseline, post-intervention, and follow-up periods. Exploratory subgroup analyses using independent t-tests will investigate differential intervention effects based on factors like age, gender, and socioeconomic status. For mediating factors (family functioning and HIV-related stigma), correlation analyses and structural equation modeling will examine relationships with primary and secondary outcomes, with appropriate techniques employed to handle missing data.

The qualitative data analysis will utilize specialized software (NVivo) for coding and organization, following Clarke and Braun [68] thematic analysis approach. This involves data familiarization, initial coding, theme development, review, and refinement. The analysis process will include multiple researchers to ensure reliability, with peer debriefing sessions and member checking to validate findings’ accuracy and trustworthiness.

## 6. Blinding

Due to the nature of the MFGT intervention, full blinding of participants and facilitators is not possible. However, outcome assessors will be blinded to group allocation to minimize assessment bias. These independent assessors will conduct evaluations without knowledge of participants’ group assignments.

## 7. Data monitoring/follow-up plan and tracking- minimizing loss to follow-up

The research team will implement a comprehensive follow-up plan to maintain high retention rates (90–95%) throughout the study. This includes collecting detailed participant contact information (including three alternative contacts), establishing monthly communication systems using mobile technology, and providing participation incentives and logistics support. A centralized data management system will track enrollment, attendance, and retention metrics, with regular monitoring of attrition rates. The study steering committee will oversee the implementation of follow-up strategies, addressing potential barriers to participation and maintaining strong relationships with participants. This systematic approach to participant tracking and retention aims to keep attrition rates below 10% while ensuring continued engagement throughout the study period [21,69,70].

## 8. Strategies to improve adherence to MFGT Intervention protocols

The MFGT intervention will employ comprehensive quality control measures to ensure protocol adherence and program fidelity. As mentioned previously, facilitators will receive initial 5-day intensive training, followed by weekly supervision meetings, regular performance feedback, and ongoing mentoring. Quality assurance measures include audio recording of sessions, random observations by supervisors, weekly attendance reviews, and monthly facilitator logs. The implementation will be monitored through standardized session outlines, protocol adherence checklists, and documentation of any adaptations. Regular team meetings will address challenges and maintain consistent intervention delivery, balancing fidelity with necessary adaptations to meet participant needs.

## 9. Ethical consideration

The study has received approval from Ghana Health Service’s institutional review boards and ethics committee (GHS-ERC:004/07/24), with written informed consent required from caregivers and written assent from adolescent participants. All documentation will be available in local languages, clearly explaining study details and participants’ rights, including withdrawal without penalty. Strict confidentiality measures include unique identification numbers, secure data storage, and limited access to research materials. The research team will monitor participant well-being, providing appropriate support and referrals as needed, with trained facilitators delivering the intervention following safety protocols. The study emphasizes beneficence through mental health and ART adherence improvements, ensures equitable participant selection, and includes oversight from an independent Data and Safety Monitoring Board to address any ethical concerns during implementation.

## 10. Patient and public involvement

Stakeholders, including adolescents living with HIV, caregivers, and healthcare providers from Lower Manya Krobo, were engaged from the study’s conceptualization through consultation meetings. Their input shaped research questions, outcome measures, intervention design, and implementation strategies. A stakeholder advisory group will guide recruitment, assess participation burden, and inform the dissemination of findings to ensure accessibility and relevance to all community members.

## 11. Current status of the study

Recruitment for the study commenced in December 2024 and is still ongoing. Till now, about 20 ALHIV and caregivers have been recruited. Preliminary mental health assessments and eligibility evaluations to determine participant qualification for the study continue to be conducted. The intervention phase is expected to begin in June 2025. In February 2025, we completed stakeholder engagement, focus group discussions, and individual interviews to adapt and modify the MFGT manual to the Ghanaian context. Recruitment and enrollment of participants is scheduled to conclude on May 31st, 2025. Randomization and allocation of participating clinics to intervention and control arms will also be finalized by May 31st, 2025. Baseline clinic assessments are slated to commence on May 15th and will continue until May 31st, 2025.

## 12. Discussion

While MFGT has shown promise in improving outcomes for adolescents living with HIV/AIDS in other settings, this intervention is yet to be wholly adapted and evaluated in the Ghanaian context. This study addresses this critical gap by tailoring the MFGT intervention to the specific needs and cultural context of Ghanaian adolescents and their families.

The three-phase study design, consisting of an adaptation phase, a pilot implementation phase, and an evaluation phase, is well-suited to ensure the cultural relevance, feasibility, and effectiveness of the MFGT intervention in the Ghanaian context. The successful implementation of this study protocol has significant implications for improving the well-being of adolescents living with HIV/AIDS in Ghana. By adapting the MFGT intervention to the local context, the research team aims to develop a culturally relevant and effective approach to addressing the mental health and ART adherence challenges faced by this vulnerable population. This tailored approach is crucial, as previous interventions developed in high-income countries may not translate seamlessly to low-and-middle-income settings [71] like Ghana. The study’s comprehensive evaluation, including quantitative assessments and qualitative data collection, will provide valuable insights into the feasibility, acceptability, and preliminary effectiveness of the adapted MFGT intervention [72,73].

The study’s strengths lie in its comprehensive approach, the integration of multiple data sources, and the strong emphasis on cultural adaptation and stakeholder engagement. Actively involving adolescents, their caregivers, healthcare providers, and policymakers throughout the research process will strengthen the relevance, acceptability, and long-term impact of the MFGT intervention [74].

However, some potential limitations should be considered. The study’s reliance on self-reported measures for the primary and secondary outcomes, such as the PHQ-9, GAD-7, and Wilson’s 3-item adherence scale, may be subject to social desirability bias or recall bias. To mitigate this, the research team may consider incorporating objective measures, such as electronic medication monitoring, to corroborate the self-reported data [75].

Additionally, the feasibility and scalability of the MFGT intervention may be influenced by the existing healthcare infrastructure, resource availability, and competing priorities within the Ghanaian healthcare system. The research team will need to closely collaborate with policymakers and healthcare administrators to identify and address potential barriers to implementation and scale-up [76].

Despite these considerations, the proposed study holds significant promise in addressing the critical mental health and ART adherence challenges faced by adolescents living with HIV/AIDS in Ghana. The findings from this study will have important implications for the broader healthcare system in Ghana. If the adapted MFGT intervention is found to be effective, it could inform the development of national guidelines and policies to support the integration of similar mental health and adherence interventions into the standard of care for adolescents living with HIV/AIDS. The cost-effectiveness analysis will also provide valuable insights to guide decisions regarding the allocation of resources and the potential for scaling up the MFGT program across the country [77].

## 13. Patient and public involvement

Patients and/or the public will be involved in the design, or conduct, or reporting or dissemination plans of this research. Please refer to the methods section for details.

## Supporting information

S1 File SPIRIT fillable checklist. (DOCX)

S2 File WHO trial registration data set. (DOCX)
