# Supplementary material for: Multiple-family group intervention programming to improve mental health of adolescents living with HIV/AIDS in Ghana: An implementation science study protocol
Source: PLoS One. 2025 Jun 12;20(6):e0325854. doi: 10.1371/journal.pone.0325854 (PMC12161527; doi:10.1371/journal.pone.0325854)
Supplement: S2 File — (DOCX) [file pone.0325854.s002.docx]

|  | **Data category** | **Information** |
| --- | --- | --- |
|  | Primary registry and trial identifying number | Clinical Trials.gov (NCT06701942) |
|  | Date of registration in primary registry | 22 November, 2024 |
|  | Secondary identifying numbers | ClinicalTrials.gov NCT06701942 |
|  | Source(s) of monetary or material support | Science for Africa Foundation |
|  | Primary sponsor | Prof. Samuel Adjorlolo  Department of Mental Health Nursing  School of Nursing and Midwifery  University of Ghana |
|  | Secondary sponsor(s) | Science for Africa Foundation |
|  | Contact for public queries | Prof. Samuel Adjorlolo  Department of Mental Health Nursing  School of Nursing and Midwifery  University of Ghana  Accra- Ghana  +233240592635  sadjorlolo@ug.edu.gh |
|  | Contact for scientific queries | Prof. Samuel Adjorlolo  Department of Mental Health Nursing  School of Nursing and Midwifery  University of Ghana  Accra- Ghana  +233240592635  sadjorlolo@ug.edu.gh |
|  | Public title | Mental Health of Adolescents living with HIV in Lower Manya Krobo District, Ghana. |
|  | Scientific title | Multiple-Family Group Intervention Programming to Improve Mental Health of Adolescents Living with HIV/AIDS in Ghana: An Implementation Science Study |
|  | Countries of recruitment | Ghana |
|  | Health condition(s) or problem(s) studied | Depression and Anxiety |
|  | Intervention(s) | Multiple Family Group Therapy: cognitive group therapy (based on 4Rs and 2Ss) that brings 8-10 families dealing with similar challenges toether to support each other and learn together with the aim to address the emotional and social challenges faced by young people living with HIV and their families.  Control group: treatment as usual |
|  | Key inclusion and exclusion criteria | *Inclusion Criteria*:  Adolescents aged 10-19 years (as per WHO definition of adolescence) with confirmed HIV-positive status, aware of their HIV status and currently receiving antiretroviral therapy (ART). Primary caregiver of an eligible adolescent participant aged 18 years or older and aware of the adolescent's HIV status. Additional criteria include adolescents and their caregivers residing in Lower Manya Krobo district, able to communicate in either English or the local language and willing to participate in group sessions.  *Exclusion criteria*  Adolescents and caregivers/families with severe cognitive impairment that would prevent participation in group activities and/ or acute psychiatric conditions requiring immediate intensive treatment, Adolescents and caregivers planning to relocate outside of Lower Manya Krobo district within the next 12 months, unable to commit to attending the majority of planned group sessions, adolescents participating in another mental health intervention study and unable to provide assent or obtain caregiver consent. Caregivers unable to provide informed consent will also be excluded |
|  | Study type | Interventional Allocation: randomized Intervention model: factorial assignment Masking: blind - outcome assessors will be blinded to group allocation, Primary purpose: prevention |
|  | Date of first enrolment | October 2024 |
|  | Sample size | Target sample size: 80; 40 adolescents living with HIV and 40 caregivers  Enrollment so far: 22 (status as of 14^th^ February 2025) |
|  | Recruitment status | Recruiting |
|  | Primary outcome(s) | Depression and Anxiety  (Time Frame: Change from baseline of the study at immediate post-intervention, 3, 6, and 12 months after the intervention |
|  | Key secondary outcomes | Adherence to antiretroviral therapy  (time frame: Change from baseline of the study at immediate post-intervention, 3 6, and 12 months after the implementation of the intervention. |
|  | Ethics Review | *The study has undergone rigorous ethics review process and has received approval from Ghana Health Service's institutional review boards and ethics committee(GHS-ERC:004/07/24)* |
|  | Completion date | *Date of expected study completion: 1^st^ December 2025*. |
|  | Summary Results | *Not yet available, Recruitment still ongoing* |
|  | IPD Statement | Plan to share IPD: Undecided - It is not yet known if there will be a plan to make IPD available. |
